# Peculiarities of the *e(y)2* Gene Evolution in Deuterostomes and Drosophilinae

**DOI:** 10.3390/ijms262110705

**Published:** 2025-11-03

**Authors:** Julia Vorontsova, Elena Belova, Anastasia Khrustaleva, Anastasia Umnova, Olga Arkova, Konstantin Boyko, Alena Nikolaeva, Oksana Maksimenko, Artem Bonchuk, Pavel Georgiev, Roman Cherezov

**Affiliations:** 1 Department of the Control of Genetic Processes, Institute of Gene Biology Russian Academy of Sciences, 34/5 Vavilov St., Moscow 119334, Russia; vjul83@mail.ru (J.V.); lenagromenko@yandex.ru (E.B.); forarkova@mail.ru (O.A.); bonchuk_a@genebiology.ru (A.B.); 2 Center for Genome Research, Institute of Gene Biology, Russian Academy of Sciences, 34/5 Vavilov St., Moscow 119334, Russia; nastia.khrust@gmail.com (A.K.); anast.popovich@gmail.com (A.U.); maksog@mail.ru (O.M.); 3 Bach Institute of Biochemistry, Research Center of Biotechnology Russian Academy of Sciences, Leninsky pr-t, 33, Bld. 2, Moscow 119071, Russia; kmb@inbi.ras.ru; 4 Complex of NBICS Technologies, National Research Center “Kurchatov Institute”, Moscow 123182, Russia; aishome@mail.ru; 5 Koltsov Institute of Developmental Biology, Russian Academy of Sciences, Moscow 119334, Russia

**Keywords:** DUB SAGA, TREX-2, *Drosophila*, e(y)2b, ENY2, retrocopy, Sgf11, gene duplication

## Abstract

Gene duplication, a major source of new genes in evolution, often occurs via reverse transcription of mRNA, leading to the integration of a retrocopy into a new genomic locus. Here, we performed an in-depth analysis of the evolutionary history of the *e(y)2* gene in Metazoa. The E(y)2 protein is a shared subunit of two highly conserved complexes involved in transcription regulation (the DUB module of the SAGA complex) and mRNA transport (TREX-2). In Deuterostomes, the *e(y)2* gene has undergone multiple independent retropositions, often giving rise to functional retrogenes. In contrast, among Protostomes, duplications of *e(y)2* were identified only in Drosophilinae and a member of the Lepidoptera family (*Manduca sexta*). In *Drosophila*, the retrocopy *e(y)2* acquired an almost ubiquitous expression pattern and compensates for the function of the parental gene in all tissues except the testes. The parental gene, *e(y)2b*, evolved a testis-specific expression pattern, lost the ability to incorporate into the DUB module, but retained nuclear envelope localization and the capacity to assemble into the TREX-2 complex. Knockout of the *D. melanogaster e(y)2b* gene resulted in reduced male fertility. Overall, our study highlights distinct evolutionary trajectories of the *e(y)2* gene in Deuterostomes and Protostomes.

## 1. Introduction

Formation of the transcriptional pre-initiation complex consisting mainly of RNA polymerase II and general transcription factors is a crucial step in eukaryotic transcription initiation [1]. One of the key complexes that regulates the transcription preinitiation complex (PIC) formation and stability is the Spt-Ada-Gcn5 acetyltransferase (SAGA) transcriptional coactivator complex, with 18 evolutionarily conserved subunits identified [2,3,4]. The SAGA complex consists of four modules: a histone acetyltransferase (HAT), a histone deubiquitinase (DUB), suppressor of Ty element (Spt) module, and TBP-associated factor (TAF) module [4,5,6]. The DUB module is responsible for the removal of ubiquitin from histone H2Bub1 in eukaryotes [7,8,9,10,11]. The degree of H2B ubiquitination is of great importance in the regulation of gene transcription in all eukaryotes and is also thought to be involved in repair, replication, and homologous recombination [12,13]. The DUB module can function independently from SAGA [14,15] or in an alternative complex called a non-stop identity complex (NIC) in *Drosophila* [16].

The *Drosophila* SAGA DUB module includes the core subunits E(y)2, Sgf11, NonStop, and dATXN7, which link the DUB module to SAGA [17]. The same organization of the DUB module has been observed in all studied organisms from yeast to humans [12,18], suggesting that the structure of E(y)2 is highly conserved and remained largely unchanged during evolution. In addition to its role in the DUB module of SAGA, E(y)2 is also a component of the highly conserved TREX-2 complex, which is involved in mRNA transport from the nucleus [18,19,20,21]. In *Drosophila*, the complex consists of Xmas-2, PCID2, and two E(y)2 subunits [17,22]. *e(y)2* is ubiquitously expressed at all developmental stages and in all tissues and is located at chrX:11,576,118–11,576,620. A paralog of *e(y)2*, named *e(y)2b* and which is located at chr3R:7,094,038–7,094,727 [23,24], has been identified in Drosophilinae. Unlike *e(y)2*, the *e(y)2b* gene contains an intron that interrupts its coding region. It has been proposed that *e(y)2b* is a parental gene, while *e(y)2* arose through retroposition of *e(y)2b* during *Drosophila* evolution [23,24].

In this study, the *e(y)2* gene was analyzed across major representatives of Metazoa. During Deuterostome evolution, duplications of *e(y)2* frequently arise through retrotranspositions, likely facilitated by the small size of its coding region. Retrocopies generally exhibit lower expression but retain the conserved protein structure of the parental gene. In contrast, *e(y)2* duplications in Protostomes are rare and found only in Drosophilinae and a single Lepidoptera species. In Drosophilinae, the parental gene *e(y)2b* encodes a protein that has lost the ability to incorporate into the SAGA DUB module but can still help form the TREX-2 complex, whereas the retrocopy-encoded protein preserves the conserved structure and enters into both the DUB module and TREX-2. Using CRISPR/Cas9, we demonstrated that knockout of *e(y)2b* reduces male fertility. Overall, these findings indicate that *e(y)2b* in *Drosophila* has acquired a specialized role in male reproductive fitness while retaining partial functional overlap with its paralog.

## 2. Results

### 2.1. Duplications of the e(y)2 Gene Arise Independently Many Times in Deuterostomia but Not in Protostomia

For an in-depth analysis of the evolutionary history of *e(y)2* (ENY2 in mammals), we conducted a search for orthologs encoding E(y)2 across Eukaryota. Data on the number of exons in these orthologous genes were obtained from NCBI using the EDirect tools (version 16.2). A complete taxonomic description of each species was retrieved from the NCBI Taxonomy Database using the R library taxize (Supplementary Figures S1–S3).

**Figure 1:**
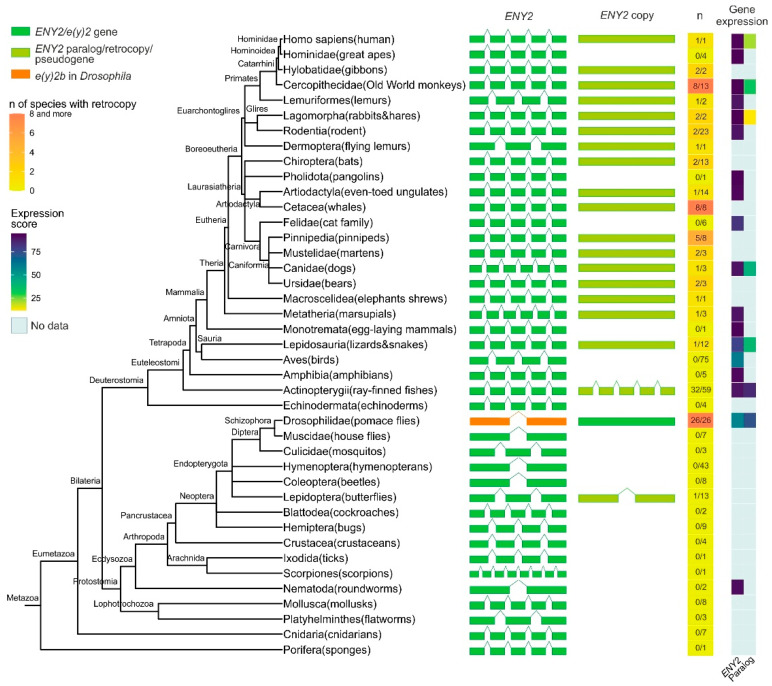
Phylogenetic tree, exon–intron gene structure, and expression levels of the *ENY2/e(y)2* gene and its paralogs across Metazoa. Exons of genes are depicted as green, olive green, and orange boxes, while introns are represented by lines connecting the boxes. In the single-column heatmap, the gradient from lemon to orange indicates the number of species with an *ENY2* retrocopy. The numbers are presented as follows: the value before the slash represents the number of species in which *ENY2* is duplicated, and the value after the slash represents the total number of analyzed species for which exon–intron structure data were available from NCBI. Gene expression data for *e(y)2* and its paralogs are displayed as a heatmap.

The analysis revealed that the E(y)2 orthologs are present in all of the examined Eukaryota species. In the majority of examined Deuterostomia species, the *e(y)2* gene displays a conserved structure comprising five exons (Figure 1, Supplementary Figures S1–S3). In many Deuterostomia taxa, we identified duplications of *e(y)2*. These duplicated copies are typically intronless, suggesting they originated via retroposition.

A duplicate of the *e(y)2* gene retaining the parental intron–exon structure, with four introns, was found exclusively in the class Actinopterygii, consistent with its emergence during the whole-genome duplication events known to have occurred in the ray-finned fish lineage [25,26,27]. Among Deuterostomia, *e(y)2* retrocopies are most frequently observed in the class Mammalia, where they are restricted to specific evolutionary lineages. Notably, although the genomic context of the parental *e(y)2* gene is highly conserved, the chromosomal localization and local genomic environment of its retrocopies vary considerably across different orders and families. No *e(y)2* duplications were detected in several taxa, including Echinodermata, Amphibia, Aves, Mammalia (Monotremata, Pholidota, Felidae, Hominidae). Interestingly, a retrocopy of *e(y)2* is present in humans (*Homo sapiens*) but is absent in other hominids. In all cases, *e(y)2* copies exhibit a high degree of amino acid sequence conservation.

In Deuterostomes, analysis of available gene expression data showed that the expression of duplicated *e(y)2* copies is generally much lower than that of the parental gene, which is highly expressed in all cells of the organism (Figure 1). This likely reflects the low probability of a retrocopy integrating under the control of a strong ubiquitous promoter without disrupting the expression of other genes. Additionally, the absence of introns in the retrocopy may reduce mRNA stability [28].

Among the studied Protostomia representatives, *e(y)2* gene duplication was observed only in Drosophilinae and in a single Lepidoptera species (*Manduca sexta*), in which two copies of the parental gene were identified (Figure 1). In Drosophilinae, the duplication event was followed by divergence in expression patterns: the parental gene, *e(y)2b*, is predominantly expressed in the testes, whereas the retrocopy exhibits strong ubiquitous expression (Figure 1). Notably, Protostomes overall display greater variability in both the number (ranging from two to four) and the positions of introns within the parental *e(y)2* gene. Interestingly, in the beetle *Onthophagus taurus*, the *e(y)2* gene contains only a single intron located in its 5′-UTR. Two non-mutually exclusive evolutionary scenarios could explain this exon–intron structure: intron loss from the coding region of the ancestral gene [29] or retrocopy replacement, in which a retrocopy fully replaced the original gene, leading to its loss, and subsequently acquired an intron in its 5′-UTR region [29].

### 2.2. Drosophila Retrocopy-Encoded E(y)2 Paralogs Retain Conserved Functions, While the Parental Gene, e(y)2b, Partially Loses It

**Figure 2:**
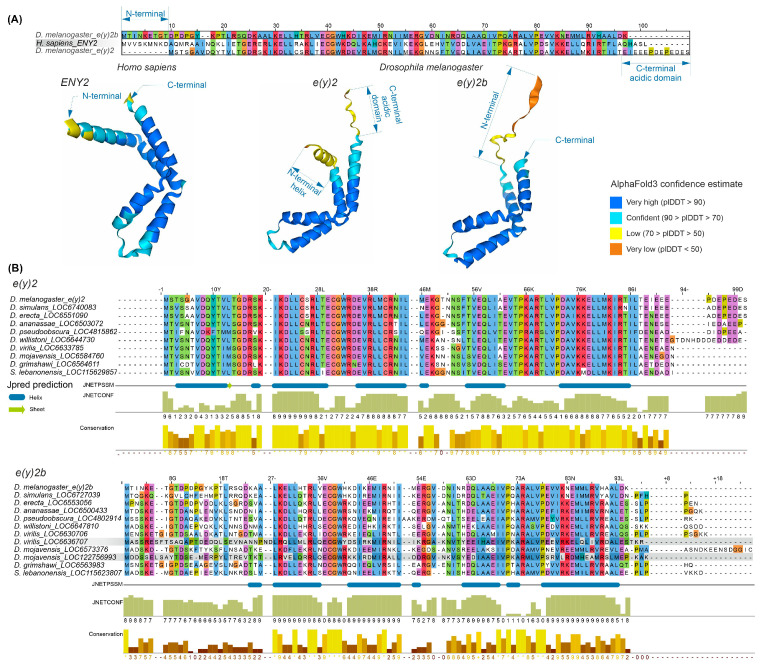
Comparison of the sequence and structure of the E(y)2 and E(y)2b proteins in *H. sapiens* and *D. melanogaster*. (**A**) The alignment of *D. melanogaster* E(y)2, *H. sapiens* E(y)2, and *D. melanogaster* E(y)2b sequences was generated using the Muscle algorithm. The lower panel shows AlphaFold3-predicted structures of E(y)2 and E(y)2b; (**B**) alignments of Drosophilinae E(y)2 and E(y)2b proteins were performed for the complete set of sequences. Below the alignment, protein secondary structure predictions were obtained using JPred4 [30] including JNetPSSM, based on the position-specific scoring matrix, and JNetCONF, a confidence estimate in which higher values indicate greater confidence. Newly identified retrocopies in *D. virilis* and *D. mojavensis* are highlighted with gray boxes, in the E(y)2b alignment. Amino acid residues are colored with Clustal X colour scheme. Sequence conservation was calculated using Jalview (version 2.11.2.5).

The E(y)2 protein has a highly conserved structure composed of five alpha helices (Supplementary Figure S4). Copies of E(y)2 in Deuterostomes retain the structural features of the parental gene. Figure 2 presents an alignment of E(y)2 protein sequences from *D. melanogaster* and humans, which primarily differ in the N-terminal region (the human protein has an extension) and the C-terminal region (the *D. melanogaster* protein contains an acidic domain). Interestingly, in *D. melanogaster*, the E(y)2b protein encoded by the parental gene differs markedly from the retrogene-encoded E(y)2 in its N-terminal region, while other regions remain highly conserved (Figure 2A). This high divergence in the N-terminal portion of E(y)2b leads to the loss of the first alpha helix in the protein (Figure 2A).

To investigate the evolutionary history of E(y)2b, we searched for its orthologs using the OrthoDB database, identifying an ortholog group comprising sequences from 47 species of the subfamily Drosophilinae. Comparison of E(y)2 and E(y)2b proteins across *Drosophila* species revealed that the N-terminal regions of E(y)2b exhibit the lowest degree of conservation (Figure 2B), whereas the remainder of the protein contains highly conserved regions homologous to E(y)2. AlphaFold3-predicted models of E(y)2b orthologs from various Drosophilinae species are shown in Supplementary Figure S5. In all analyzed species, including *S. lebanonensis* (genus *Scaptodrosophila*), E(y)2b lacks the N-terminal alpha helix. Notably, despite substantial divergence in amino acid sequences, the secondary structure of E(y)2b orthologs remains highly similar, indicating that its structure and function have been maintained throughout evolutionary history. We calculated the ratio of non-synonymous to synonymous substitutions (dN/dS) to determine the mode of selection acting on E(y)2 and E(y)2b in *Drosophila* (Supplementary Table S1). The analysis revealed that both proteins are under strong negative selection, suggesting a slow evolutionary rate. However, in *Drosophila*, E(y)2b exhibits a higher evolutionary rate compared to E(y)2 (Supplementary Figure S6).

**Figure 3:**
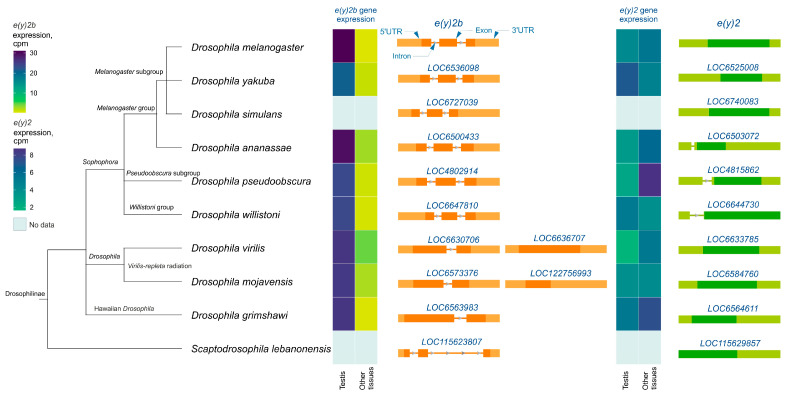
The *e(y)2* and *e(y)2b* gene exon–intron structures and their mRNA expression levels in Drosophilinae species. Orthologs of *e(y)2b* are depicted in orange, and those of *e(y)2* in green. The phylogenetic relationships among species are according to NCBI Taxonomy Database. Gene expression data, measured in counts per million (cpm), were obtained from the GEP UCSC Genome Browser [31] and visualized using heatmaps with distinct scales.

Analysis of *e(y)2* and *e(y)2b* gene structures in Drosophilinae revealed that in all members of the *Sophophora* genus, the *e(y)2b* gene contains two introns (Figure 3), whereas in members of the *Drosophila* genus, it contains only one intron. The ancestral gene structure likely consisted of two introns and three exons, as observed in *S. lebanonensis*, with species of the *Drosophila* genus having lost the first intron. Interestingly, in *D. virilis* and *D. mojavensis*, a second retrocopy of *e(y)2b* has emerged, suggesting that this duplication occurred after the origin of the *e(y)2* retrogene. The *e(y)2* gene generally consists of a single exon, except in the *ananassae*, *pseudoobscura*, and *willistoni* subgroups, where an intron has been acquired within the 5′-UTR region. The *e(y)2* retrocopy was likely already present in the common ancestor of Drosophilinae, as it is found in all representatives of the genus *Drosophila* and the genus *Scaptodrosophila* located in a conserved syntenic region. This retrocopy is absent in other dipterans (Figure 3).

Analysis of available RNA-seq data from various Drosophilinae species revealed that *e(y)2b* is predominantly expressed in the testes across all studied *Drosophila* species (Figure 3). In contrast, the *e(y)2* gene exhibits high ubiquitous expression, although it is slightly downregulated in the testes relative to other tissues in certain species, including *D*. *yakuba*, *D*. *willistoni*, and *D*. *mojavensis* (Figure 3).

We also analyzed the protein structure of E(y)2 orthologs from *Onthophagus taurus* and *Manduca sexta* using AlphaFold3 (Supplementary Figure S7). The E(y)2 protein from *O. taurus* exhibits a conserved five alpha-helix structure, including an N-terminal alpha helix, suggesting that this gene represents the ancestral form rather than a retrocopy, despite lacking introns in its coding region. In *M. sexta*, we identified two E(y)2 paralogs of the parental gene (LOC115447136). Their encoding genes (LOC115439941 and LOC115439942) are adjacent on chromosome 27, each containing two exons, similar to the parental gene on chromosome 23. Both of these paralogs lack the N-terminal alpha helix, and the protein encoded by LOC115439942 additionally acquires a beta-sheet near its C-terminus (Supplementary Figure S7). The absence of the first alpha helix renders these proteins structurally similar to E(y)2b in Drosophilinae species.

### 2.3. E(y)2b Interacts with Sgf11 but Loses Its Ability to Enter the DUB Module of SAGA

**Figure 4:**
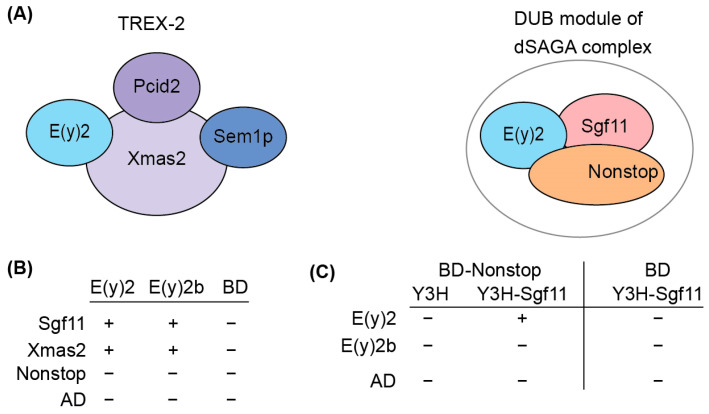
Determination of the interactions between E(y)2 and E(y)2b with NonStop, Sgf11, and Xmas2 by Y2H and Y3H assays. (**A**) Schematic of the *Drosophila* TREX-2 complex and the DUB module of SAGA; (**B**) interactions of *Drosophila* E(y)2 and E(y)2b with Sgf11, Xmas, and NonStop detected using yeast two-hybrid screening; BD—GAL4 DNA binding domain, AD—GAL4-Activating domain; (**C**) interactions of *Drosophila* E(y)2 and E(y)2b with Sgf11 and NonStop assessed using yeast three-hybrid screening. Growth assay results for the yeast plates are provided in Supplementary Table S2.

To elucidate the functional role of E(y)2b, we investigated its ability to incorporate into the DUB and TREX-2 complexes (Figure 4A). In the DUB complex, E(y)2 interacts with Sgf11 and NonStop. Based on the known structure of the yeast DUB complex [19,32], alpha-helix 1 and alpha-helices 2–5 of E(y)2 mediate interactions with NonStop and Sgf11, respectively. In *Drosophila*, the TREX-2 complex comprises Xmas-2, PCID2, and two ENY2 subunits [17,22], with E(y)2 interacting specifically with Xmas-2, which forms the backbone of the complex. To assess whether E(y)2b can participate in DUB and TREX-2 complex formation, we compared the interactions of E(y)2 and E(y)2b with Sgf11 and NonStop (DUB complex) and Xmas-2 (TREX-2 complex) using yeast two-hybrid (Y2H) and yeast three-hybrid (Y3H) assays (Figure 4B).

**Figure 5:**
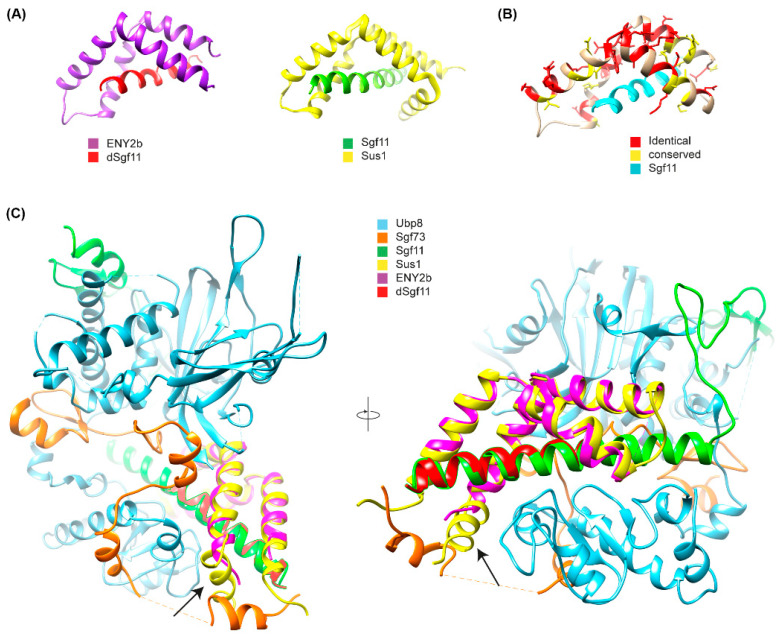
Crystal structure of the E(y)2b/Sgf11 complex. (**A**) Crystal structure of the *Drosophila* E(y)2b–dSgf11 complex (left) and yeast Sus1–Sgf11 complex (right), PDB: 3KIK [32]; (**B**) Conservation of residues between E(y)2 and E(y)2b mapped onto the E(y)2b structure; (**C**) Overlay of the E(y)2b–dSgf11 structure with the yeast DUB complex structure (PDB: 3M99) [34]. The arrow points at the missing alpha-1-helix. Figures were prepared with UCSF Chimera (version 1.10.1) [35].

We detected interactions of both E(y)2 and E(y)2b with Xmas-2, indicating their ability to incorporate into the TREX-2 complex (Figure 4B, Supplementary Table S2). Both proteins also interacted with Sgf11 in the Y2H assay. However, neither interacted with NonStop, consistent with the yeast SAGA DUB module structure [33]. To assess whether E(y)2b can enter the DUB module like E(y)2, we performed a Y3H assay (Figure 5C). In the absence of Sgf11, neither protein interacted with Nonstop. When Sgf11 was co-expressed, only E(y)2 formed a complex with NonStop, indicating formation of the E(y)2/Sgf11/NonStop complex, whereas E(y)2b/Sgf11 failed to form a complex with NonStop. These results demonstrate that E(y)2b has lost the ability to enter the SAGA DUB module but retains the capacity to interact with Xmas-2.

To gain further insight into the mechanism of complex formation, we determined the crystal structure of the E(y)2b/Sgf11 complex (Figure 5, Supplementary Figure S8).

The overall structure is similar to the previously reported structure of yeast Sus1 in complex with Sgf11 [32] (Figure 5A,B), with one key difference: it lacks alpha-helix 1, which mediates the interaction with the UBP module of NonStop and is therefore critical for the association of Sgf11/E(y)2 with the DUB module of the SAGA complex (Figure 5C). The total buried surface area upon complex formation is approximately 16% for the E(y)2b molecule and 41% for Sgf11, indicating a strong interaction. Complex formation is dominated by hydrophobic interactions, as reflected by the large solvation energy gain of –12 kcal/mol upon complex formation. The complex is further stabilized by a hydrogen bond (L4–H90) and salt bridges (D17–R74 and D18–K28). We also performed AlphaFold2-Multimer modeling [36] of the *Drosophila* DUB complex, which was predicted with high confidence and recapitulates the same interaction mechanism between E(y)2–Sgf11 and NonStop as observed in the yeast DUB complex structure [34,37] (Figure 5A). In this model, alpha-helix-1 of E(y)2 binds to alpha-3 of the UBP-finger of NonStop, forming a mostly hydrophobic interface (V12, L13 of E(y)2) reinforced by a hydrogen bond involving the side chain of Q9 (Figure 5B). Additional polar interactions between alpha-helix-2 of E(y)2 and the catalytic domain of NonStop (mediated by D22 and E30; the latter is conserved in E(y)2b) contribute to complex stabilization and proper spatial arrangement of the subunits (Figure 5C). Together, these results confirm that E(y)2b interacts with Sgf11 but does not participate in the DUB module.

### 2.4. The e(y)2b Gene Increases Male Fertility

**Figure 6:**
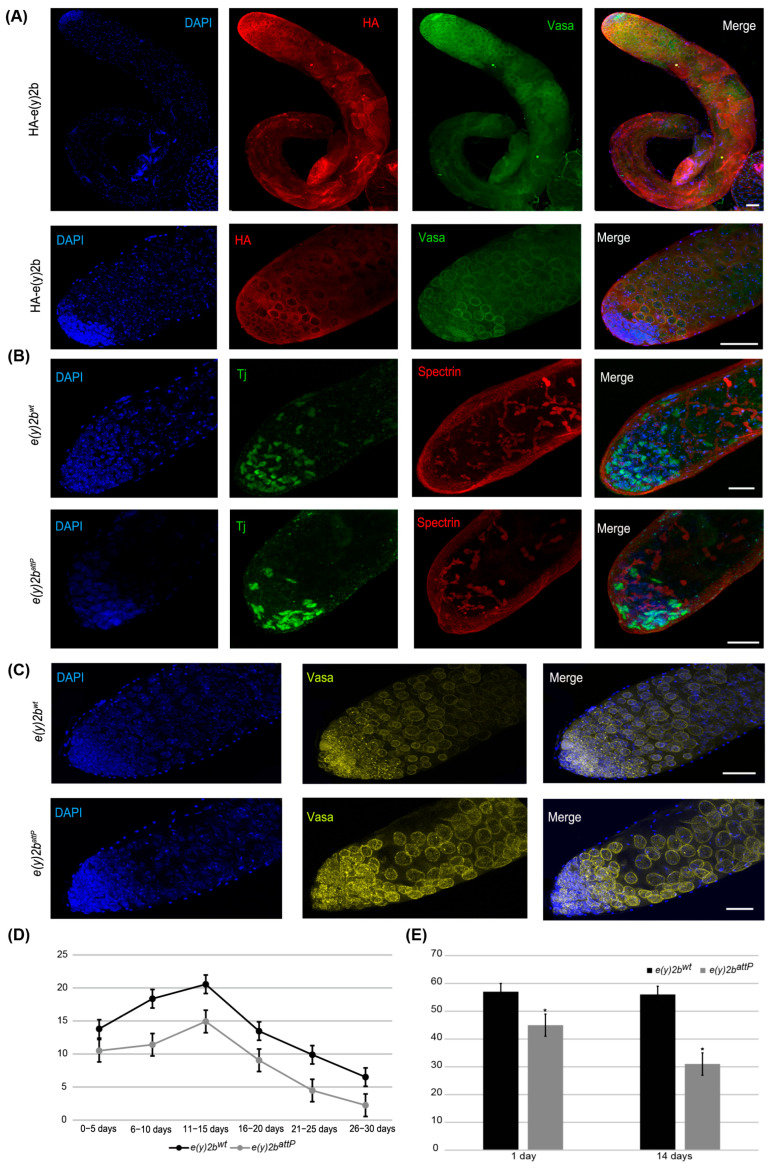
E(y)2b localizes predominantly in the apical zone of the testis and is essential for maintaining the proper cellular environment in the testis stem cell niche. (**A**) Immunostaining of testes from HA–E(y)2b males with anti-HA (red), anti-Vasa (green), and DAPI (blue). Upper panel: whole testis, lower panel: the apical zone of testis; (**B**,**C**) testes from 14-day-old *e(y)2b^attP^* males exhibit a reduced number of cyst stem cells, gonialblasts, and spermatogonia in the apical zone compared to those of *e(y)2b^wt^* males. Testes were stained with anti-Tj (green) to mark early somatic cells, anti-Spectrin (red) to label the fusome, anti-Vasa (yellow) for germline cells, and DAPI (blue) for DNA; (**D**) fertility assays show that *e(y)2b^attP^* males produce fewer offspring than *e(y)2b^wt^* males; (**E**) quantification of Tj-positive cells in testes of 1- and 14-day-old *e(y)2b^attP^* (*n* = 12) and *e(y)2b^wt^* (*n* = 12) males. The data are presented as the means ± SEM. *—*p* < 0.05 between the number of Tj-positive cells in testes of *e(y)2b^wt^* and *e(y)2^attP^* males (two-sample *t* test) Scale bar, 50 µm.

To study the in vivo E(y)2b functions, we deleted the *e(y)2b* gene in the *y^1^w^1118^* line (Supplementary Figure S9) using CRISPR/Cas9 technology described in the Materials and Methods [38,39]. To generate a loss-of-function allele, 731 bps of the *e(y)2b* gene, including its promoter and proximal coding region, were replaced with an attP site and a 3P3:dsRed reporter flanked by loxP sites, creating the *e(y)2b^attP^* allele (Supplementary Figure S9). The 3P3:dsRed reporter was subsequently excised via Cre/lox recombination between the loxP sites. *e(y)2b^attP^* flies displayed normal viability but reduced male fertility. To confirm the fertility disorders in *e(y)2b^attP^* males, a control line was generated by reintegrating the *e(y)2b* gene at the attP site in the *e(y)2b^attP^* line (Supplementary Figure S9). As a result, the *e(y)2b* gene was restored in the *e(y)2b^attP^* background (*e(y)2b^wt^*). The fertility of *e(y)2b^attP^* and *e(y)2b^wt^* males was then compared in three independent experiments. *e(y)2b^attP^* males produced fewer offspring on average than *e(y)2b^wt^* males, demonstrating that E(y)2b is required for male fertility (Figure 6D).

To determine the precise localization of E(y)2b in the male gonad, a transgenic line was generated in which the full *e(y)2b* gene, tagged at the N-terminus with a 3×HA epitope, was inserted into the attP site of the *e(y)2b^attP^* line (Supplementary Figure S9). The HA–E(y)2b protein localizes predominantly to the apical zone of the testes (Figure 6A). The apical zone of the *Drosophila* testis is a stem cell niche that serves as the primary site of cellular proliferation. It contains a central cluster of hub cells surrounded by germline stem cells and somatic cyst stem cells. This region is essential for spermatogenesis: germline stem cells produce gonialblasts, the first differentiating germ cells, while somatic cyst stem cells differentiate to generate cyst cells that support the developing germline cells throughout the testis [40,41]. Staining of the testes with Vasa, to label perinuclear granules of germline cells, and Spectrin, to visualize the fusome extending through interconnected spermatogonia and spermatocytes, revealed that HA–E(y)2b localizes to the nuclear envelope of gonialblasts, spermatogonia, and spermatocytes (Figure 6A).

To investigate the cause of reduced fertility in *e(y)2b^attP^* males, testes from 14-day-old males were stained for Vasa and Tj, a marker of somatic cyst stem cells and early cyst cells that surround gonialblasts and spermatogonia in the niche [42]. This analysis revealed a reduction in the number of Tj-expressing somatic cells, indicating a corresponding decrease in cyst stem cells, gonialblasts, and spermatogonia within the apical zone (Figure 6B,C,E).

Thus, E(y)2b is essential for maintaining the proper cellular environment within the testis stem cell niche. Its localization at the nuclear envelope of early germ cells suggests a role in their function or regulation. The *e(y)2b^attP^* mutation leads to a reduction in niche cells, disrupting spermatogenesis and consequently reducing male fertility.

## 3. Discussion

Our analysis revealed that *e(y)2* copies in Deuterostomia arise primarily through independent retroposition events within each phylogenetic lineage. For example, although *e(y)2* retrocopies are widespread among mammals, their emergence in the common ancestor of Boreoeutheria appears unlikely. This conclusion is supported by two key observations: first, the retrocopy is absent in several representatives of the superorder, and second, the genomic environments flanking the retrocopies vary considerably across different orders and families. A conserved arrangement of flanking genes is observed only within lower-ranking taxa, such as the infraorder Cetacea and specific families of primates and carnivores. These observations suggest that *e(y)2* gene duplications in Deuterostomes arose repeatedly and independently across different taxa.

In contrast to Deuterostomes, duplication of the *e(y)2* gene in Protostomes has occurred only in Drosophilinae via retroposition and in *M. sexta* (Lepidoptera), presumably through a DNA-mediated mechanism. In *Drosophila*, the parental gene (*e(y)2b*) has partially lost its function and structural integrity. In *M. sexta*, the duplicated *e(y)2* copies also encode proteins with a truncated first alpha helix, preventing these variants from participating in DUB module formation. We hypothesize that the loss of the alpha helix in *M. sexta* duplicates may result from relaxed evolutionary constraints under conditions of high, potentially redundant expression of all three orthologs.

It can be hypothesized that increased *e(y)2* expression does not confer an adaptive advantage in Protostomes and is therefore selected against. Instead, duplicated copies in *M. sexta* or the parental gene in Drosophilinae encode E(y)2 paralogs that have lost the ability to form the DUB module.

Expression data for E(y)2 and E(y)2b in testes suggest that high levels of the Sgf11/NonStop/E(y)2 DUB module may be detrimental, leading to slight downregulation of *e(y)2* in these organs in some *Drosophila* species. Moreover, E(y)2b may compete with E(y)2 for binding to Sgf11, thus reducing DUB complex assembly efficiency. At the same time, E(y)2b retains the ability to interact with Xmas-2, indicating its participation in TREX-2 complex formation. The TREX-2 complex, which functions in mRNA export, is predominantly localized in the nucleoplasm but is also concentrated at the nuclear envelope within the nuclear pore complex (NPC) [21,43]. As a component of TREX-2, E(y)2 is predominantly co-localized at the NPC. Our data indicate that E(y)2b is also localized to the nuclear envelope in gonialblasts, spermatogonia, and spermatocytes, supporting its role in the TREX-2 complex. TREX-2 is essential for shuttling mRNP particles from transcription sites to the NPC [21,44], a process likely critical for spermatogenesis. Thus, in germline cells of the testes, an increased abundance of TREX-2 complexes may be required for efficient mRNA transport, whereas a high concentration of the DUB module could negatively affect the specialized expression of certain gene groups. Indeed, knockout of *e(y)2b* in male testes leads to a decrease in the number of somatic niche cells expressing Tj (Traffic jam) and their associated cyst stem cells, gonialblasts, and spermatogonia, ultimately reducing male fertility. Despite substantial divergence in the amino acid sequence, the secondary structure of E(y)2b is conserved among Drosophilinae species, supporting the proposal that in all Drosophilinae, E(y)2b participates exclusively in TREX-2 complex formation.

The evolutionary history of *E(y)2b* and *E(y)2* in Drosophilinae provides a rare example that challenges existing models of retrogene evolution: the “Out of the X” and “Out of the Testis” hypotheses. The “Out of the X” model proposes that genes on the X chromosome are the primary sources of retroposition events, with their retrocopies being transposed to autosomes [45,46,47]. The “Out of the Testis” model posits that newly emerged genes, regardless of origin, most often exhibit highest expression in the testes, a pattern facilitated by the genomic environment and regulatory landscape of this tissue [48,49,50]. In this case, the parental gene *e(y)2b* is autosomal, whereas its retrocopy has transposed to the X chromosome. Despite its X-chromosome location, the *e(y)2* retrogene is ubiquitously expressed, in contrast to the testis-specific expression of *e(y)2b*.

We propose that this exception arises from the successful integration of the *e(y)2* retrocopy under the control of a ubiquitous promoter, which allowed it to establish and maintain its evolutionarily favored ubiquitous expression across all cell types. During subsequent evolution, the protein encoded by the parental gene lost alpha-helix 1 and became specialized for functions exclusively in the testes. As a result, in most *Drosophila* species, the *e(y)2b* gene exhibits a testis-specific expression pattern.

Thus, duplicated copies of *e(y)2* in Deuterostomes are generally not favored by selection. In contrast, in Drosophilinae, we observe a striking example of retrocopy adaptation: the retrocopy acquires a ubiquitous expression pattern and retains all functions of the parental gene, while the parental gene evolves a specialized, testis-specific role that is maintained by selection due to its contribution to reproductive fitness.

## 4. Materials and Methods

### 4.1. Bioinformatics Analysis

A primary search for orthologs of the *D. melanogaster* E(y)2 and E(y)2b proteins was performed in Eukaryota using the OrthoDB [51] and EGGNOG [52] databases, and the corresponding protein sequences were exported. The total number of the identified orthologs from OrthoDB was 1289 and 241 from the EGGNOG. To retrieve the NCBI IDs and exon count data from the NCBI database for each orthologous protein, E-Utilities’ EDirect tools were used [53]. The amino acid sequences of E(y)2 and E(y)2b orthologs were aligned with the Muscle algorithm in JalView 2.11.2.5 [54]. The resulting multiple sequence alignments (MSAs) of full-length protein were manually trimmed and then processed automatically with trimAl [55] (all positions in the alignment with a gap frequency of more than 30% were removed). Phylogenetic relationships between the orthologs were inferred using maximum likelihood (ML) tree reconstruction in IQ-TREE 2.0.3 [56]. The most suitable mutation models for amino acid substitutions were previously determined in ModelFinder module implemented in IQ-TREE. The taxonomic hierarchy was reconstructed according to the NCBI Taxonomy Database using Taxize [57]. Gene expression patterns across multiple animal species were retrieved from the Bgee database [58]. Relative gene expression data for *Drosophila* species was acquired from the GEP UCSC Genome Browser [31]. Protein secondary structures were predicted using the JPred4 module in JalView 2.11.2.5. Molecular structures were predicted with AlphaFold3 [59]. The ratio of non-synonymous to synonymous substitutions (dN/dS) was calculated for each ortholog pair of Drosophila E(y)2 and E(y)2b using the CodeML program in the PAML package (version 4.10.9) [60]. Coding sequences (CDS) for each ortholog were extracted from their respective genomes and used to convert a protein multiple sequence alignment into a codon alignment by PAL2NAL [61]. A dN/dS ratio of <1, =1, and >1 was interpreted as evidence of negative selection, neutral evolution, and positive selection, respectively.

### 4.2. Protein Expression and Purification

*E. coli* BL21(DE)3 co-transformed with vectors expressing full-length untagged E(y)2b and dSgf1140-68 with TEV-cleavable GST-tag were grown at +37 °C in 5L of LB media until reaching OD of 0.6, then were cooled to +18 °C and induced with 1 mM IPTG overnight at +18 °C. The following procedures were performed at +4 °C. All buffers were degassed to prevent protein and DTT oxidation. Cells were pelleted, resuspended in lysis buffer (20 mM Tris (pH 7.4), 150 mM NaCl, 20 mM KCl, 5 mM MgSO_4_, 0.01 mM ZnCl_2_, 10% *w*/*w* glycerol, 0.1% NP40, and 5 mM DTT), containing protease inhibitors, disrupted with high-pressure homogenizer (Microfluidics), centrifuged at 20,000× *g* for 1 h and applied to 5 mL of pre-equilibrated gluthatione-resin (Pierce). The resin was washed with a lysis buffer containing 500 mM NaCl and subjected to TEV-cleavage overnight at +4 °C at a constant rotation in a buffer containing 20 mM Tris (pH 8.0), 200 mM NaCl, 5 mM Na-Citrate, and 1 mM DTT. Flowthrough containing cleaved proteins was collected, concentrated and the complex was purified with size-exclusion chromatography with Superdex 10/300GL (GE Healthcare) in 20 mM Tris (pH 8.0), 200 mM NaCl, and 1 mM DTT.

### 4.3. Crystallization and Data Collection

Initial crystallization screening was performed on a Rigaku robotic system (Rigaku Americas Corporation, Houston, TX, USA) using 96-well VDX plates (Hampton Research, Aliso Viejo, CA, USA) and commercial crystallization screens from Hampton Research (Aliso Viejo, CA, USA) and Molecular Dimensions Inc. (Holland, OH, USA) by the “hanging drop” vapor diffusion method. A 10 mg/mL of the complex in 20 mM HEPES buffer pH 8.0 containing 50 mM NaCl, 100 μM PLP, and 1 mM DTT was mixed with the crystallization solution in the ratios 1:1, 1:2 (0.1 μL drop volume), and 2:1 (0.2 μL drop volume). The volume of the precipitant solution in the reservoir was 50 µL. The initial crystallization hit was observed under the following conditions: 0.1 M MES pH 6.0; 20% isopropanol, 22% PEG 2000 MME at 288 K. Further optimization of crystals growth was done using the “hanging drop” vapor-diffusion method in 24-well VDX plates (Hampton Research, Aliso Viejo, CA, USA). The drop volume was increased to 3 μL, and the volume of the precipitant solution to 500 μL. Crystals were briefly soaked in a mother liquor containing 20% glycerol immediately before diffraction data collection and flash-frozen in liquid nitrogen. Datasets were collected at 100 K at MASSIF-3 beamline (Grenoble, France). The dataset was indexed, integrated, and scaled using the XDS package (https://xds.mr.mpg.de/, accessed on 31 October 2025) [62]. Space group was initially suggested by Pointless [63] as C222, but it turned out that crystal was twinned and the real space group was C2 (Table S2).

### 4.4. Structure Solution and Refinement

Detailed refinement statistics are shown at Table S3. The structure of the complex was solved by the molecular replacement method using the MOLREP program (Version 11.0) [64] and the model prepared with AlphaFOLD2 [36]. Two copies of the complex were found in an asymmetric unit. A total of 17 residues at the N-terminus have no electron density in e(y)2b molecules. In Sgf11 molecules, only the first 27 residues have electron density (24 residues for the second molecule from the asymmetric unit).

The refinement was carried out using the REFMAC5 program of the CCP4 suite (version 5.5). The visual inspection of electron density maps and the manual rebuilding of the model were carried out using the COOT interactive graphics program (version 1.1.x) [65]. The isotropic B-factor, NCS and hydrogen atoms in fixed positions were used during the refinement.

### 4.5. Yeast Two-Hybrid and Three-Hybrid Assays (Y2H, Y3H)

A yeast two-hybrid assay (Y2H) was carried out using *Saccharomyces cerevisiae* PJ69-4A strain (*MATa trp1–901 leu2*–*3*, *112 ura3*–*52 his3*–*200 gal4*∆ *gal80*∆ *LYS2*::*GAL1*-*HIS3 GAL2*-*ADE2 met2*::*GAL7*-*lacZ*) with plasmids and protocols from Clontech (Clontech, Mountain View, CA, USA). Briefly, for growth assays, plasmids encoding fusions with the GAL4 activation domain (AD) and DNA-binding domain (BD) were co-transformed into yeast strain pJ69-4A by the lithium acetate method, according to the manufacturer’s instructions. For the yeast three-hybrid (Y3H) assay, the pY3H plasmid was additionally used for co-transformation. Transformed cells were plated on selective medium lacking leucine and tryptophan (Y2H) or lacking leucine, tryptophan and uracil (Y3H), and incubated at 30 °C for 3 days. Subsequently, colonies were streaked onto a selective medium without leucine, tryptophan and histidine (Y2H) or leucine, tryptophan, uracil and histidine (Y3H), and incubated at 30 °C for 2–3 days. Each assay was performed in three independent replicates.

### 4.6. Plasmids Used in the Yeast Two-Hybrid and Three-Hybrid Assays

The CDS of *Drosophila melanogaster* E(y)2, E(y)2b, Sgf11, Xmas2 and NonStop were PCR-amplified using corresponding cDNA as a template and in-frame cloned into either a GAL4 DNA-binding or activation domain-containing vector (pGBT9 (Clontech, Mountain View, CA, USA) or pGAD424 (Clontech, Mountain View, CA, USA), respectively) using restriction sites *EcoR*I and *Sal*I. For Y3H assays, we used plasmid pY3H and corresponding cDNAs were subcloned using the same restriction sites.

### 4.7. Generation of the e(y)2b^attP^ Platform Using CRISPR/Cas9

*Drosophila* strains were grown at 25 °C on standard wheat meal–yeast–sugar–agar medium. We used the fly CRISPR Optimal Target Finder tool (http://targetfinder.flycrispr.neuro.brown.edu/, accessed on 25 October 2025) to design a CRISPR target sequence for the 5′ and 3′ ends of the *e(y)2b* gene. The sgRNAs were cloned into a pCR vector derived from the pCFD4-U6:1_U6:3tandemgRNAs plasmid (Addgene #49411) using *Bbs*I restriction sites. The 5′ and 3′ flanking homology arms surrounding the CRISPR/Cas9 target sites were cloned into a homologous recombination plasmid (pHR), flanking a dsRed reporter cassette that was itself flanked by loxP sites. The Cas9 nuclease was supplied via a helper plasmid (Addgene #62209). A mixture of these plasmids (10:1—pHR:pCR, concentration 300 ng/µL) was injected into *y^1^w^1118^* embryos. Potential genome editing events were detected by dsRed fluorescence. The resulting flies were crossed with *y^1^w^1118^*; TM6B/Sb balancer line. For *Cre*-*loxP*-mediated DNA, fragment excision flies were crossed with a recombinase-expressing line (*y^1^w^1118^*; *Kr^If−^*^1^*/Cyo*, *P[Cre w+]DH1; MKRS/TM6B*). Excision was confirmed by PCR analysis.

### 4.8. Generation of Transgenic Lines

The constructs were assembled in a pBluescriptSK vector backbone and contained genetic elements in the following order: [attB]-[loxP]-[5′genomic region including promoter and 5′UTR-*e(y)2b^wt^* or 3xHA-*e(y)2b*-3′genomic region including 3′UTR]-[SV40polyA]-[eGFP-3xP3 promoter] (Figure S9). *e(y)2b^wt^* was generated by PCR amplification of genomic DNA with Q5 High-Fidelity DNA Polymerase (NEB, Ipswich, MA, USA). 5′UTR-3xHA-*e(y)2b*-3′UTR region was generated by overlap extension polymerase chain reaction (PCR) using the primers listed in Supplementary Table S4 and was verified by sequencing. The resulting constructs, along with a plasmid expressing the *ϕC31* recombinase (Addgene plasmid #26290) were microinjected into embryos of the *y^+^w^1^; e(y)2b^attP^* dsRed platform described above. Successful integrations into the attP site of the platform were selected on the basis of expression of the mini-white reporter in the eyes [66]. Subsequently, the reporter and plasmid body sequence were excised by *Cre*/*lox*-mediated recombination between the *loxP* sites. All crosses were conducted using the *y^1^w^1118^*; *TM6B/Sb* balancer line.

### 4.9. Fertility Tests

To assess age-dependent male fertility, newly eclosed males (≤8–12 h post-eclosion) (*n* = 25 for both the experimental and control groups) were collected and housed individually in food vials. Each male was initially mated with three 3–5-day-old virgin wild-type females. After five days, the males were transferred to fresh vials, and the original vials were retained for 12 days to quantify viable offspring. The males were then remated with a new cohort of three 3–5-day-old virgin wild-type females. This mating cycle was repeated until the males were 25–30 days old.

Fertility was quantified as the number of offspring produced per male during each mating interval. All experiments were conducted at 25 °C in a temperature-controlled incubator under a light–dark cycle simulating natural diurnal conditions.

### 4.10. Histology, Immunofluorescence Staining and Microscopy

Testes from adult fly tissues were dissected in 1xPBS (Phosphate-Buffered Saline) and fixed for 15 min in 1xPBS with 4% formaldehyde. After three washes 10 min each in 1xPBS with 0.2% Triton X-100 (PBST), samples were blocked using 3% BSA in PBST for 1 h, then incubated overnight at 4 °C with primary antibodies in blocking solution. The following primary antibodies were used: mouse anti-α Spectrin (1:50) (Developmental Studies Hybridoma Bank, Iowa City, IA, USA), rat anti-Vasa (1:100) (Developmental Studies Hybridoma Bank, Iowa, USA), guinea-pig polyclonal anti-Tj (1:5000) [42]. The secondary antibodies were anti-mouse, anti-rat and anti-guinea pig conjugated to Alexa Fluor 488, 594, or 633 (Invitrogen, Carlsbad, CA, USA). DNA was stained with DAPI (AppliChem, Darmstadt, Germany). Preparations were mounted in Vectashield (Vector Laboratories, Newark, NJ, USA). Images were acquired with a Leica STELLARIS 5 confocal microscope. Confocal images were processed using ImageJ FIJI (https://imagej.net/software/fiji/downloads, last accessed 20 October 2025) [67] and figures were assembled using Photoshop CS4 software (version 20.0.3). To determine the number of Tj-positive cells in the testis, we used Leica LAS software (version 4.5.0.25531) to generate Z-stacks of optical sections of the apical zone in 0.5 μm steps. ImageJ FIJI software (https://imagej.net/software/fiji/downloads, accessed on 20 October 2025) was used to go through the Z-stack to quantify all Tj-positive in a testis.

### 4.11. Statistical Analysis

Statistical analysis on cell number of Tj-positive cells in testes of *e(y)2b^wt^* and *e(y)2b^attP^* males was executed using a two-sample *t*-test by JASP (Version 0.95.3).

## Data Availability

All data generated or analyzed during this study are included in this published article and its Supplementary Materials.

## Supplementary Materials

The following supporting information can be downloaded at https://www.mdpi.com/article/10.3390/ijms262110705/s1. Reference [68] has been mentioned in text.
