# Supplementary material for: Peculiarities of the e(y)2 Gene Evolution in Deuterostomes and Drosophilinae
Source: Int J Mol Sci. 2025 Nov 3;26(21):10705. doi: 10.3390/ijms262110705 (PMC12611072; doi:10.3390/ijms262110705)
Supplement: Supplementary file 1 [file ijms-26-10705-s001.zip › Link for downloading the original confocal images.pdf]

The original images for confocal microscopy can be downloaded at:
https://disk.yandex.ru/d/JAJyCSVMa8pSCg
