# Supplementary material for: Peculiarities of the e(y)2 Gene Evolution in Deuterostomes and Drosophilinae
Source: Int J Mol Sci. 2025 Nov 3;26(21):10705. doi: 10.3390/ijms262110705 (PMC12611072; doi:10.3390/ijms262110705)
Supplement: Supplementary file 1 [file ijms-26-10705-s001.zip › ENY2B MS Supplementary_Figures and tables MDPI 03 11.pdf]

Supplementary information for

Peculiarities of the *e(y)2* gene evolution in Deuterostomes and Drosophilinae

Julia Vorontsova[1&], Elena Belova[1&], Anastasia Khrustaleva[2&], Anastasia Umnova[2], Olga Arkova[1], Konstantin Boyko[3], Alena Nikolaeva[4], Oksana Maksimenko[2], Artem Bonchuk[1], Pavel Georgiev[1*], Roman Cherezov[1,5*]

[1] Department of the Control of Genetic Processes, Institute of Gene Biology Russian Academy of Sciences, 34/5 Vavilov St., Moscow 119334, Russia;
[2] Center for Genome Research, Institute of Gene Biology, Russian Academy of Sciences, 34/5 Vavilov St., Moscow 119334, Russia;
[3] Bach Institute of Biochemistry, Research Center of Biotechnology Russian Academy of Sciences, Leninsky pr-t, 33, Bld. 2, Moscow, 119071, Russia;
[4] Kurchatov Complex of NBICS-Technologies, National Research Centre "Kurchatov Institute", 123182 Moscow, Russia;
[5] Koltsov Institute of Developmental Biology, Russian Academy of Sciences, Moscow 119334, Russia

& Equal contribution

*Correspondence: georgiev_p@mail.ru (P.G.), ro-tcherezov@yandex.ru (R.C.)

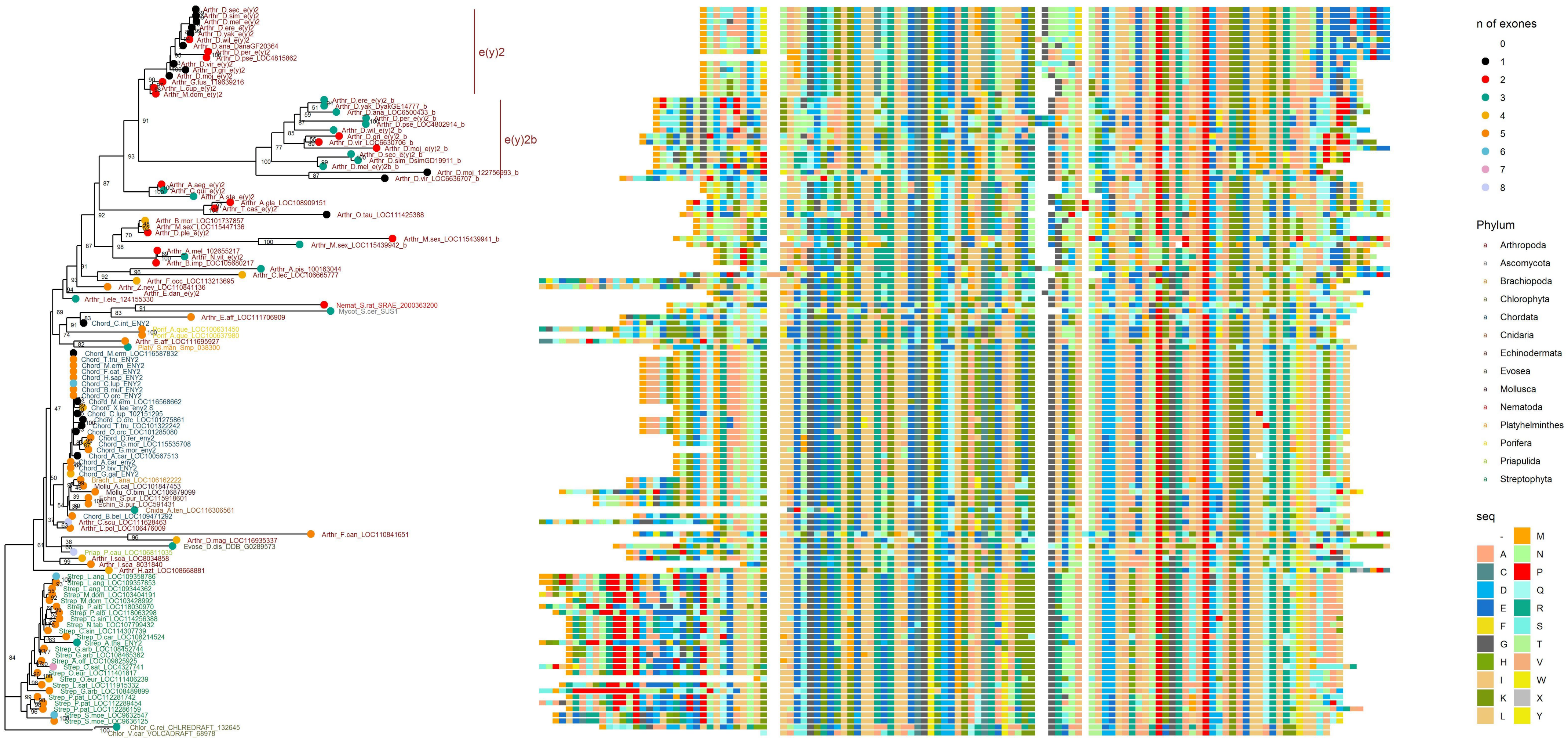

Figure S1. **A maximum likelihood (ML) phylogenetic tree and the alignment of E(y)2 protein orthologs from *D. melanogaster* within the Eukaryota domain.** Bootstrap support values are shown at the nodes. The circles at the terminal nodes are color-coded to represent the number of exons in the corresponding gene, and the branch labels are colored according to their phylum/division.

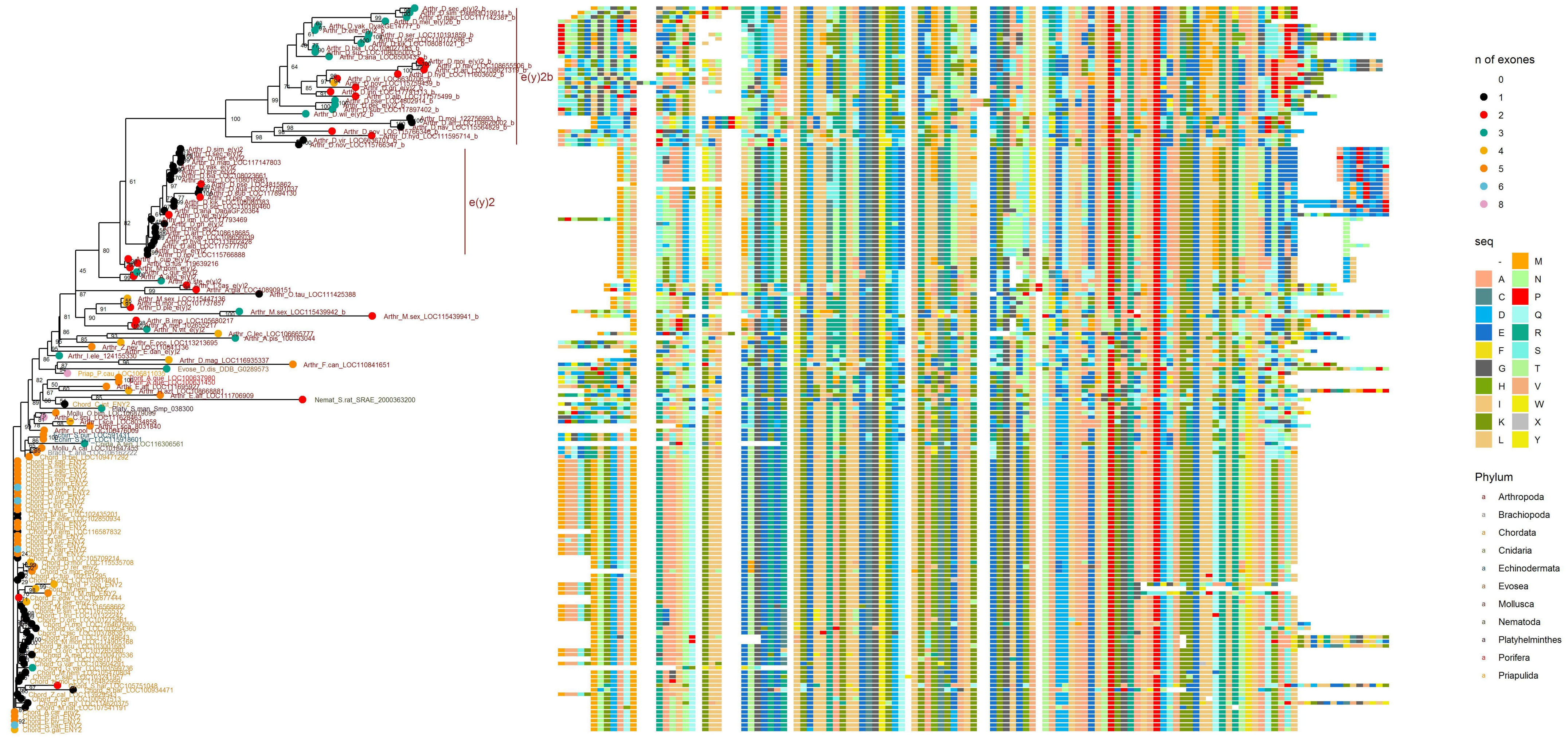

Figure S2. **A maximum likelihood (ML) phylogenetic tree and protein alignment of D. melanogaster E(y)2 orthologs within the Metazoa and Evosea.** Bootstrap support values are shown at the nodes. The circles at the terminal nodes are color-coded to represent the number of exons in the corresponding gene, and the branch labels are colored according to their phylum/division.

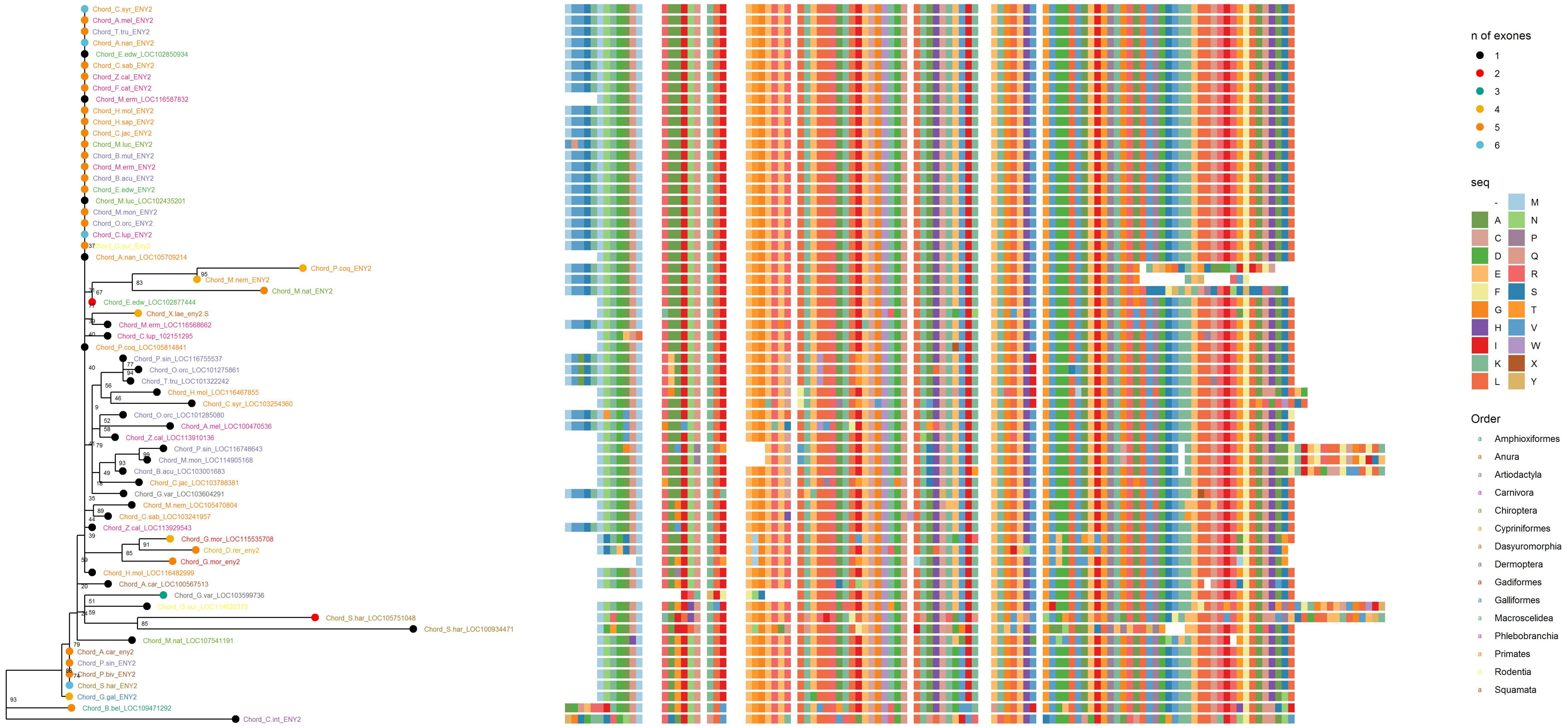

Figure S3. A maximum likelihood (ML) phylogenetic tree and protein alignment of chordate E(y)2 orthologs. Bootstrap support values are indicated at the nodes. Terminal nodes are marked with circles color-coded to represent the number of exons in the corresponding gene. The branch labels are colored according to their taxonomic order.

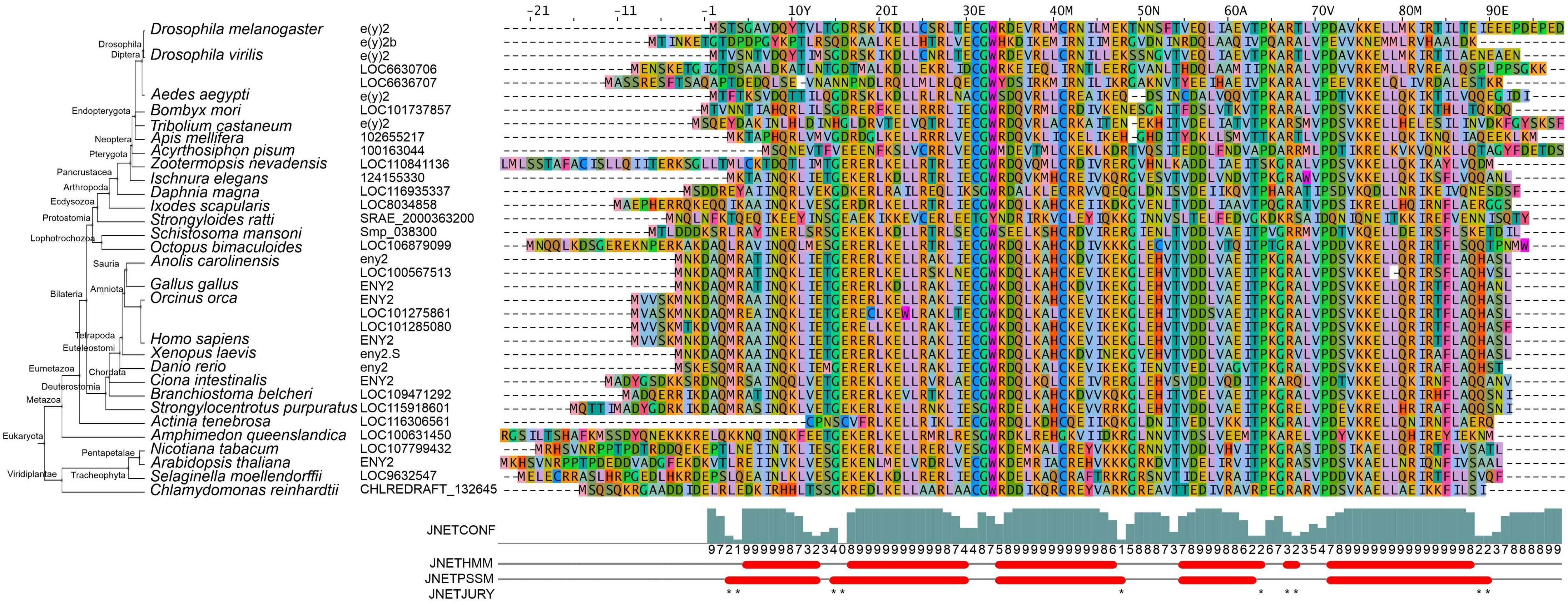

Figure S4. ENY2/E(y)2b and E(y)2 protein sequences in taxonomically different organisms showed conservative structure of the ENY2 protein across Eukaryota. Below the alignment Jpred [1] secondary structure prediction is displayed. The JNETCONF track displays confidence scores, JNETHMM and JNETPSSM tracks display predicted alpha-helices with HMM profile based prediction and PSSM based prediction, helices are marked as red tubes. JNETJURY was invoked to rationalise significantly different primary predictions.

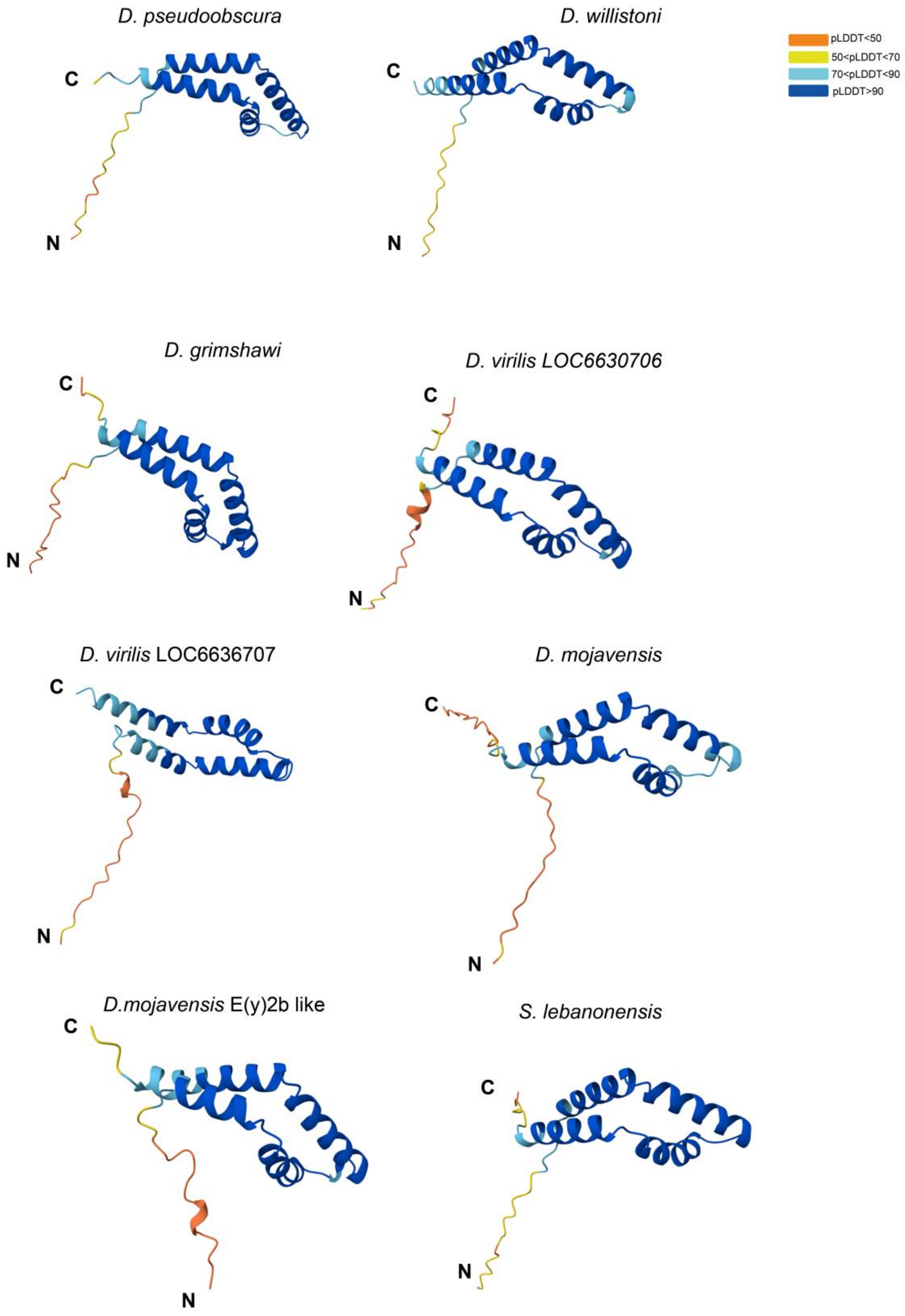

Figure S5. The AlphaFold3-predicted structures of E(y)2b orthologs in *Drosophilinae.* The predicted AlphaFold3 structures are colored according to the per-residue confidence score (pLDDT; range 0-100). Residues with higher scores represent regions of higher prediction confidence.

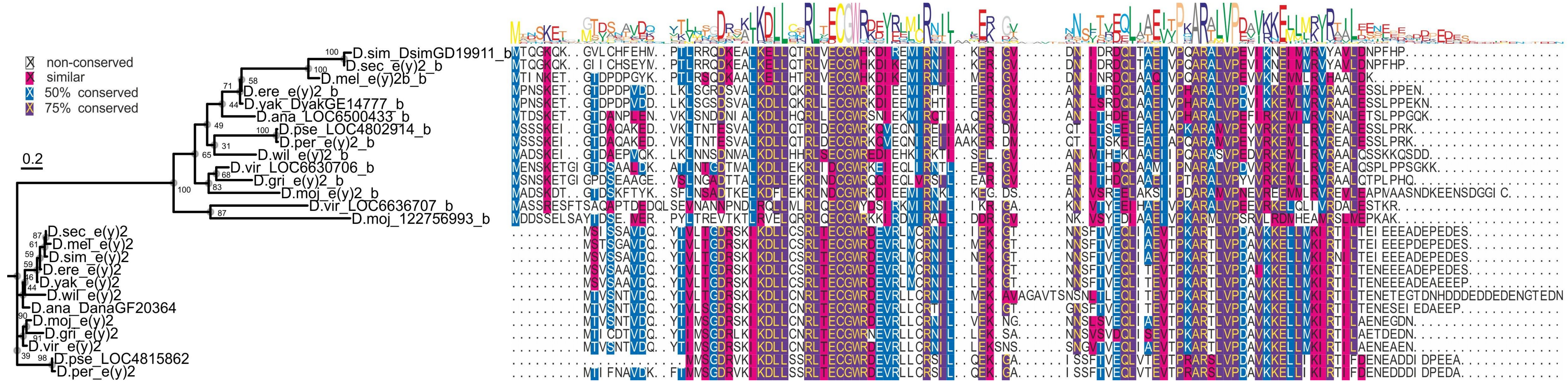

Figure S6. A maximum likelihood (ML) phylogenetic tree and the alignment of *Drosophila* E(y)2 and E(y)2b protein orthologs.

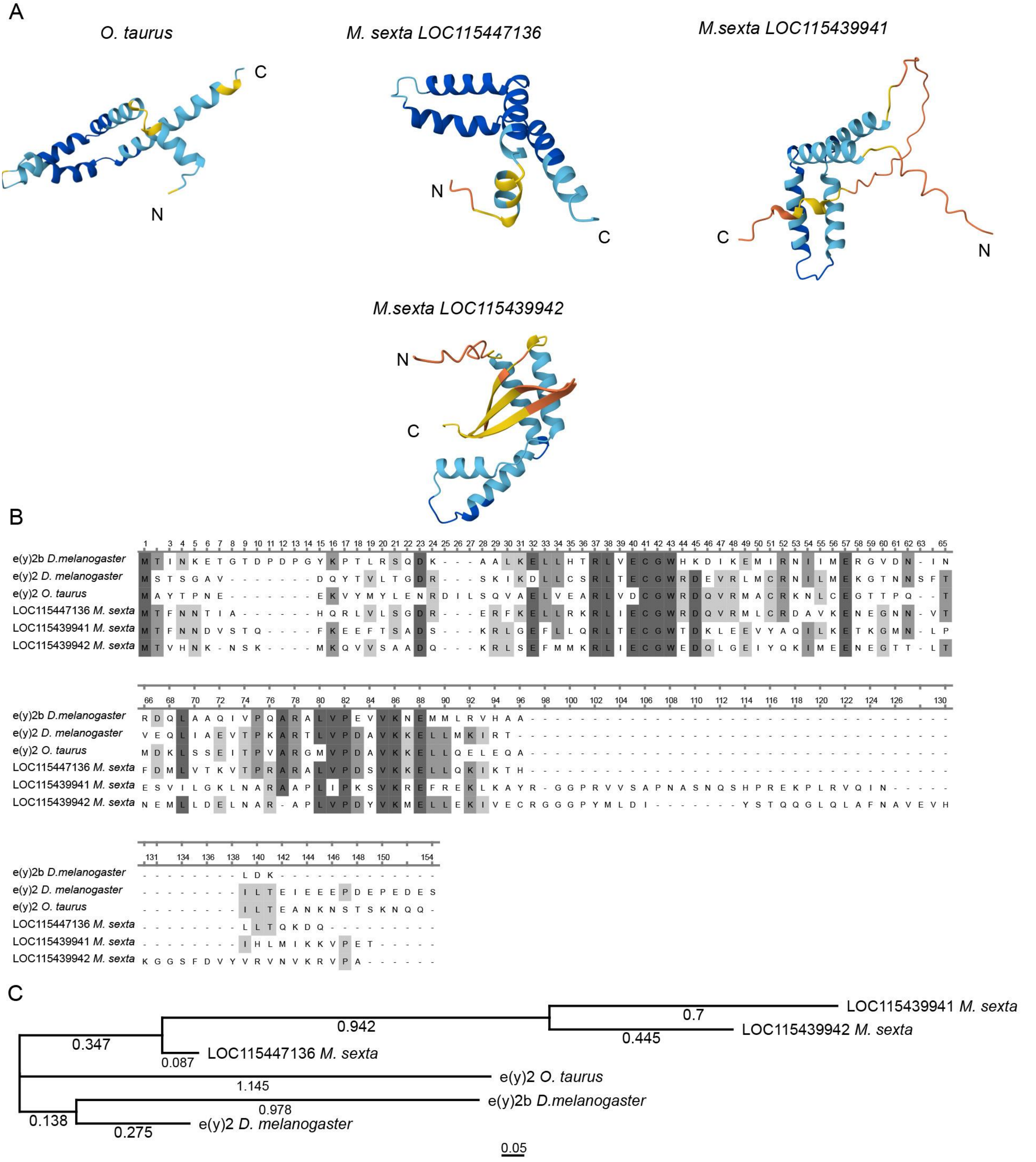

Figure S7. Analysis of E(y)2 proteins and their paralogs in *O. Taurus* and *M. sexta*. (**A**) The AlphaFold3-predicted structures of *E(y)2* orthologs in *O. Taurus* and *M. sexta*. The predicted AlphaFold3 structures are colored as described in Fig. S5; (**B**) Protein alignment of *E(y)2* orthologs from *D. melanogaster*, *O. taurus* and *M. sexta*; (**C**) A maximum likelihood (ML) phylogenetic tree of E(y)2 and its orthologs in *D. melanogaster*, *O. taurus* and *M. sexta*.

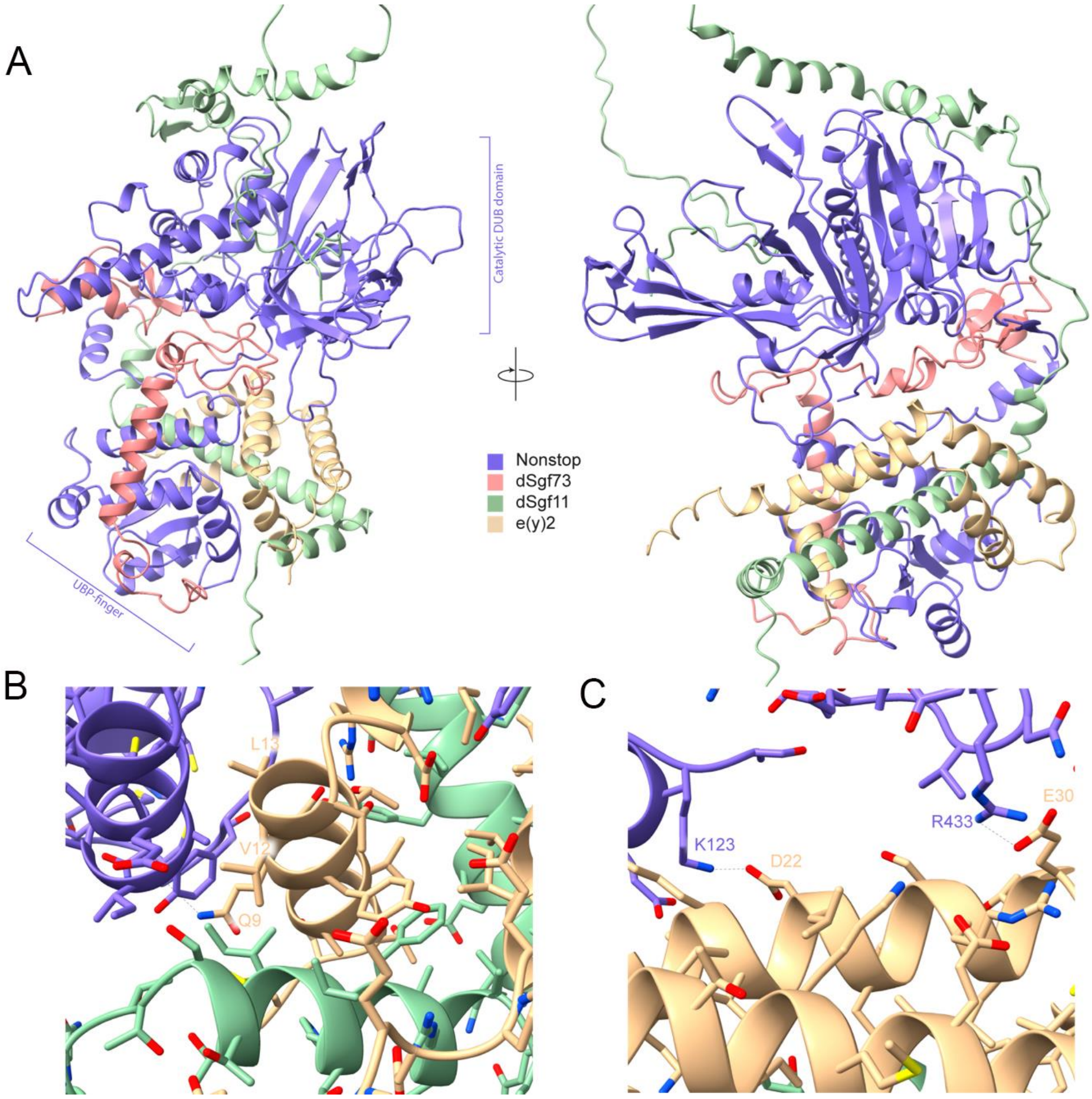

Figure S8. Structure of Drosophila Nonstop complex.

(**A**) AlphaFold-Multimer model of Drosophila Nonstop complex. Close-up view of the interaction of alpha 1-helix of E(y)2 with UBP-finger of Nonstop (**B**) and catalytic domain (**C**). Figures prepared with UCSF Chimera X [2].

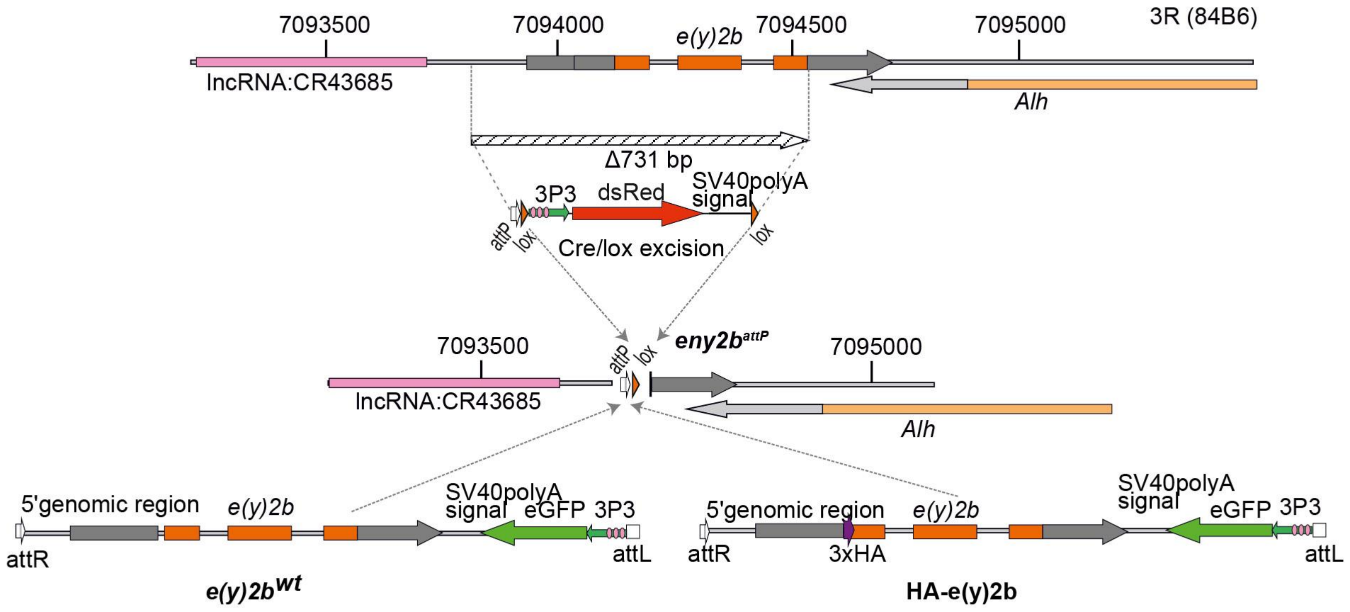

Figure S9. CRISPR/Cas9-mediated editing of the *e(Y)2b* gene and generation of 3xHA-e(y)2b and e(y)^{wt} constructs. The *e(y)2b^{attP}* line was obtained through the substitution of a 731bp region (dm6 coordinates chr3R:7093808-7094550) with *attP* site and the *dsRed* gene, flanked by *loxP* sites. The *3P3:dsRed* reporter was deleted by Cre/lox recombination between the *loxP* sites. Constructs bearing e(y)2b^{wt} and 3xHA-tagged e(y)2b were created using pBluescriptSK-based vector with 3xP3-EGFP marker as described in Materials and methods and integrated into attP site in eny2b^{attp} line using φC31-based integration system [3].

Table S1. The ratio of non-synonymous to synonymous substitutions (*dN*/*dS*) calculated for *Drosophila* E(y)2 and E(y)2b

Species 1		Species 2	S	N	dN	dS	dN/dS	Log-Likelihood
D.vir_E(y)2b	-	D.mel_E(y)2b	60,6	170,4	0,3122	1,8102	0,1725	-512,072959
D.gri_E(y)2b	-	D.mel_E(y)2b	61,1	169,9	0,334	2,1021	0,1589	-525,664469
D.pse_E(y)2b	-	D.vir_E(y)2b	59	172	0,3344	4,9121	0,0681	-531,575943
D.wil_E(y)2b	-	D.vir_E(y)2b	57	174	0,241	2,4758	0,0973	-508,449053
D.wil_E(y)2b	-	D.pse_E(y)2b	58,5	172,5	0,3362	2,5986	0,1294	-532,400635
D.mel_E(y)2	-	D.wil_E(y)2	60,4	170,6	0,0768	2,6315	0,0292	-449,724792
D.vir_E(y)2	-	D.mel_E(y)2	42,5	188,5	0,0776	1,7043	0,0455	-422,92761
D.vir_E(y)2	-	D.gri_E(y)2	45,8	185,2	0,047	1,3502	0,0348	-414,955164
D.pse_E(y)2	-	D.gri_E(y)2	56,4	174,6	0,1382	2,0621	0,067	-469,654102
D.pse_E(y)2	-	D.vir_E(y)2	52,1	178,9	0,1444	4,695	0,0308	-462,265693

Table S2. Detection of interaction between E(y)2 and E(y)2b and NonStop, Sgf11 and Xmas2 by Y2H and Y3H assays.

1) BD-Eny2 / AD-Sgf11<br>2) BD-Eny2 / AD-Xmas2<br>3) BD-Eny2 / AD-Nonstop<br>4) BD-Eny2 / AD	5) BD-Eny2b / AD-Sgf11<br>6) BD-Eny2b / AD-Xmas2<br>7) BD-Eny2b / AD-Nonstop<br>8) BD-Eny2b / AD	9) BD / AD-Sgf11<br>10) BD / AD-Xmas2<br>11) BD / AD-Nonstop<br>12) BD / AD
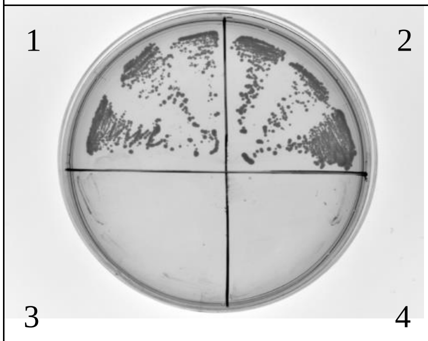	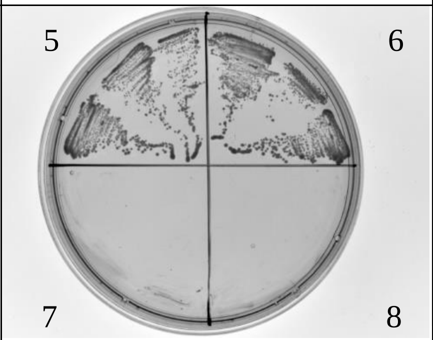	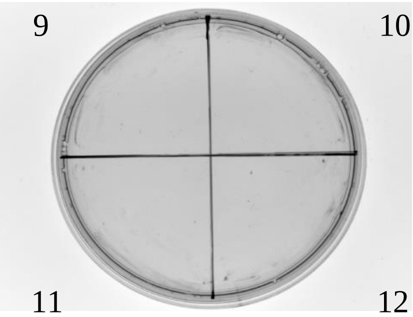
13) BD-Nonstop / AD-Eny2 / Y3H<br>14) BD-Nonstop / AD-Eny2b / Y3H<br>15) BD-Nonstop / AD / Y3H<br>16) BD-Nonstop / AD-Eny2 / Y3H-Sgf11	17) BD-Nonstop / AD-Eny2b / Y3H-Sgf11<br>18) BD-Nonstop / AD / Y3H-Sgf11<br>19) BD / AD-Eny2 / Y3H-Sgf11<br>20) BD / AD-Eny2b / Y3H-Sgf11	21) BD / AD / Y3H-Sgf11
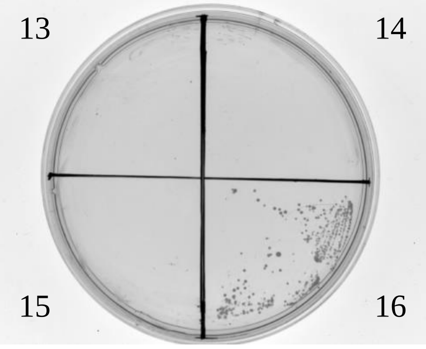	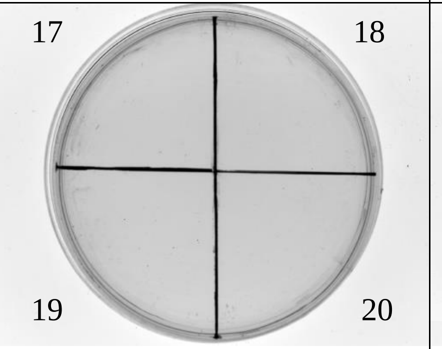	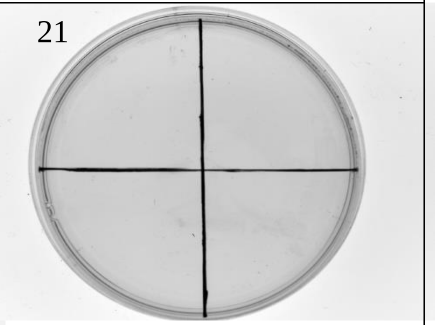

Table S3. Data collection and processing. Values for the outer shell are given in parentheses.

**Data collection**	
Diffraction source	MASSIF-3 beamline (ESRF)
Detector	Pilatus
Wavelength (Å)	0.97
Temperature (K)	100
Space group	C2
a, *b*, *c* (Å)	45.96 57.55 98.89
α, β, γ (°)	90.00 90.03 90.00
Resolution range (Å)	18.75-2.55 (2.66-2.25)
Completeness (%)	94.6 (98.9)
Redundancy	2.7 (2.8)
$\langle I/\sigma(I)\rangle$	4.9 (1.0)
R_{meas} (%)	7.6 (138.7)
$CC_{1/2}$ (%)	99.9 (41.5)
**Refinement**	
$R_{\rm work}$ (%)	21.4
$R_{\rm free}$ (%)	26.2
**No. of non-H atoms**	
Protein	1532
Others	0
**R.m.s. deviations**	
Bonds (Å)	0.01
Angles (°)	2.34
**Ramachandran plot (%)**	
Favored	84.4
Outliers	4.0
**Average *B* factors (Å²)**	
Protein	100.38
Others	0
**MolProbity score**	2.19
**PDB ID**	9JUA

Table S4. Primers used in the study

Primers for the yeast two-hybrid, three-hybrid assays and (5' to 3' direction)	
Eny2_d	TTGAATTCATGAGCACTTCCGGCGC
Eny2_ r	TTGTCGACTTAGGATTCGTCCTCTGG
E(y)2b_d	TTGAATTCATGACAATAAACAAGGAAA
E(y)2b_r	TTGTCGACTTACTTATCGAGGGCGGCGT
Sgf11_d	TTGAATTCATGTCTGCAGCCAACATGCCGAC
Sgf11_r	TTGTCGACCTAAAAGGTTTTGCCATTGTTCTTC
Sgf11_shot_r	TTGTCGACCTAGTCCATTGGCTTTTTGGCC
Xmas2_d	TTGAATTCCTGAAACTGGAACAGTTGTTTTTTG
Xmas2_r	TTGTCGACTTAGCCTAGTTCGTAGATGGATTGC
Nonstop_d	TTGAATTCATGTCCGAGACGGGTTGTC
Nonstop_r	TTGTCGACTTACTCGTATTCCAGCACATT
Primers for CRISPR/Cas9 and generation of transgenic lines (5′-3′ direction)	
5′ey2b_guide	GAGAAGACCTTTCG GTCACGGGACAAAGGTTACT GTTTTAGAGCTAGAAATAGCAAG
3′ey2b_guide	GAGAAGACCTAAAC ATGGGCAGTCACTACTTATC GACGTTAAATTGAAAATAGGTC
5′reg_ey2b_d	TTGAATTCCAATCACTGGAGCATCCC
5′reg_ey2b_r	TTCATATGGTCCCGTGACAGAAATTCA
3′reg_ey2b_d	TTAGATCTGCCCATCGGTTCCATCCT
3′reg_ey2b_r	TTCTCGAGGCACTGCCATGTTTCAAGC
5UTR_ey2B_H111_d	TTAAGCTTTGGATTTGAAACGAGTTCCG
5UTR_ey2B_r	GAGATCCATGGTTCGAGTTTTAATTCGATT

3HA_ey2B _d	ACTCGAACCATGGATCTCCACCGCGG
3HA_ey2B _r	TTTATTGTCATGCCATGAGCAGCGTAATC
3UTR_ey2B_d	GCTCATGGCATGACAATAAACAAGGAAACG
3UTR_ey2B_R1_r	TTGAATTCAGAACACGTGATTTTTTAAGG
5e2bXI	ACACTCGAGCAAATCCATCGAGGACTTG
3e2bH3	CTGAAGCTTGCACTGCCATGTTTCAAGC

Supplementary references

1. Drozdetskiy, A.; Cole, C.; Procter, J.; Barton, G.J. JPred4: A Protein Secondary Structure Prediction Server. *Nucleic Acids Res* **2015**, *43*, W389–W394, doi:10.1093/nar/gkv332.
2. Pettersen, E.F.; Goddard, T.D.; Huang, C.C.; Meng, E.C.; Couch, G.S.; Croll, T.I.; Morris, J.H.; Ferrin, T.E. UCSF ChimeraX: Structure Visualization for Researchers, Educators, and Developers. *Protein Science* **2021**, *30*, 70–82, doi:10.1002/pro.3943.
3. Bischof, J.; Maeda, R.K.; Hediger, M.; Karch, F.; Basler, K. An Optimized Transgenesis System for Drosophila Using Germ-Line-Specific φC31 Integrases. *PNAS* **2007**, *104*, 3312–3317.
